# Comparable antigen-specific T cell responses in vaccinees with diverse humoral immune responses after the primary and booster BBIBP-CorV vaccination

**DOI:** 10.1080/22221751.2022.2130101

**Published:** 2022-10-26

**Authors:** Dong Wei, Yingying Chen, Xiaoqi Yu, Yang-dian Lai, Wenxin Xu, Ping Ji, Zhitao Yang, Erzhen Chen, Xinxin Zhang, Ying Wang

**Affiliations:** a Department of Infectious Diseases, Research Laboratory of Clinical Virology, Ruijin Hospital, Shanghai Jiao Tong University School of Medicine, Shanghai, People’s Republic of China; b Department of Immunology and Microbiology, Key Laboratory of Cell Differentiation and Apoptosis of Chinese Ministry of Education, Shanghai Jiaotong University School of Medicine, Shanghai Institute of Immunology, Shanghai, People’s Republic of China; c Department of Emergency, Ruijin Hospital, Shanghai Jiao Tong University School of Medicine, Shanghai, People’s Republic of China; d Clinical Research Center, Ruijin Hospital, Shanghai Jiao Tong University School of Medicine, Shanghai, People’s Republic of China; e Shanghai Key Laboratory of Emergency Prevention, Diagnosis and Treatment of Respiratory Infectious Diseases, Shanghai, People’s Republic of China; f Key Laboratory of Parasite and Vector Biology, Ministry of Health, School of Global Health, Chinese Center for Tropical Diseases Research, Shanghai Jiao Tong University School of Medicine, Shanghai, People’s Republic of China

**Keywords:** COVID-19 vaccines, T cell responses, B cell responses, SARS-CoV-2, Omicron

## Abstract

BBIBP-CorV exerts efficient protection against SARS-CoV-2 infection. However, waning vaccine-induced humoral immune responses after two-dose vaccination have significantly undermined durable immuno-protection. In this study, we have demonstrated that although anti-spike (S) antibody responses in BBIBP-CorV vaccinees exhibited three serotypes after 6 months, including *de novo* sero-negative, sero-positive, and sero-decay features, S-specific interferon-γ release as well as Th1 cytokine production in CD4^+^ and CD8^+^ T cells were comparable, especially in vaccinees without detectable neutralizing antibodies. Notably, regardless of dramatic increases in humoral immunity after booster vaccination, T cell responses targeting S protein from either wild type or Omicron remained stable before and after booster vaccination in all three serotype vaccinees. No severe cases were observed even in the sero-decay group during the Omicron epidemic in Shanghai. Our results thus illustrate that unlike fluctuating humoral responses, viral-specific T cell responses are extremely stable after booster vaccination. Sustained T cell responses might be dedicated to the rapid restoration of antibody responses after booster vaccination.

## Introduction

The Coronavirus Disease 2019 (COVID-19) caused by severe acute respiratory syndrome coronavirus 2 (SARS-CoV-2) continues to be a huge burden on public health worldwide [1]. Up to now, World Health Organization (WHO) has announced 350 candidate SARS-CoV-2 vaccines in clinical and pre-clinical development [2–4]. It is well addressed that all types of SARS-CoV-2 vaccines trigger humoral immune responses with increased levels of antigen-specific IgG with neutralizing activity. The induction of neutralizing antibody (nAb) is the key criterion for the evaluation of vaccine efficacy. Vaccine-induced spike protein-specific nAb (S-nAb) is also recognized as an independent fact to prevent the severity of COVID-19 once infected by either wild-type or SARS-CoV-2 variants [5].

However, real-world data reveal that vaccine-induced antibody titres as well as neutralizing activity wane over time in most of the available SARS-CoV-2 vaccines, especially in elders and those with clinical risk groups [6,7]. In most cases S-nAb levels decrease after 6 months of vaccination [8]. In our previous work, we reported that antibodies against SARS-CoV-2 existed only in 78.42% (229/292) BBIBP-CorV vaccinees at 8–9 months after two-dose BBIBP-CorV vaccination with an 8.8-fold decrease in antibody titres. 81.85% of the participants lost their S-nAb responses [9]. In addition, there are certain proportions of BBIBP-CorV vaccinees with no detectable S-nAbs after vaccination [10]. Limited duration of humoral immunity as well as non-responsiveness after full-course vaccination implies the impairment of long-term humoral immunity of available SARS-CoV-2 vaccines, making it a great challenge to explore the relevant mechanisms and subsequent improvement.

In this study, we enrolled 84 BBIBP-CorV-vaccinated subjects from the previous study [10] to determine humoral and T cell responses at different vaccination stages. Based on S-nAb levels at 1 and 6 months after two-dose BBIBP-CorV vaccination, the vaccinees were subgrouped into seronegative (Vac-neg, with *de novo* deficiency in S-nAb), seropositive with sustained S-nAb at 6 months (Vac-Pos/SS) and seropositive with decayed S-nAb at 6 months (Vac-Pos/SD). Interestingly, three groups exhibited comparable S protein-specific T cell responses to wild-type (WT) SARS-CoV-2 and B.1.1.529 (Omicron) variant of concern (VOC) demonstrated by antigen-specific interferon (IFN)- γ release and cytokine production in CD4^+^ and CD8^+^ T cells. More importantly, booster vaccination with a third dose of BBIBP-CorV remains an efficient way to restore the activity of humoral immunity against SARS-CoV-2 WT and Omicron VOC with stable specific T cell immune responses.

## Materials and methods

### Study design

The objective of this study was to investigate the vaccine-induced cellular immunity against SARS-CoV-2 and variants in vaccinees without detectable neutralizing antibodies. A prospective cohort study of health care workers (HCWs) in Shanghai Ruijin Hospital was initiated in 2021 with a focus on the immuno-protection of BBIBP-CorV (NCT04795414). From January 2021, HCWs were offered vaccination with 2 doses of BBIBP-CorV at an interval of 21 days, and 84 participants were invited for the follow-up study in which immune profiles upon vaccination were studied. From September 2021, 38 HCWs in the study were invited for a follow-up study after booster vaccination with the homologous vaccine. We also enrolled 12 individuals without vaccination as control (non-Vac). Venous blood samples were collected and separated to obtain serum and peripheral blood mononuclear cells (PBMCs). Serum samples were used to measure neutralizing antibodies. PBMCs were used for interferon-γ releasing assay and flow cytometry. Based on S-nAb levels at 1 and 6 months after two-dose BBIBP-CorV vaccination, the participants were subgrouped into seronegative (Vac-neg, with *de novo* deficiency in S-nAb), seropositive with sustained S-nAb at 6 months (Vac-Pos/SS) and seropositive with decayed S-nAb at 6 months (Vac-Pos/SD). All participants had no documented history of SARS-CoV-2 infection. This study was approved by the Medical Ethical Committee at Shanghai Ruijin Hospital (RJHKY2021-12). The study adheres to the principles of the Declaration of Helsinki and written informed consent was obtained from every participant. This study was unblinded and not randomized.

### Sample collection

Serum from all vaccinated participants was obtained 28 days (28dpp) and 6 months (6mpp) after the second dose, while peripheral mononuclear cells were obtained only at 6mpp after the second dose. Additional serum and PBMC samples were obtained at 28 days (28dpb) after booster vaccination. Participants of this study had no history of previous SARS-CoV-2 infection, as confirmed by the absence of spike/nucleocapsid-specific antibodies before vaccination at baseline.

### Serum and PBMC isolation

Serum was collected from whole blood in 5-ml tubes without anticoagulant, centrifugated at 2500 rpm for 15 min, aliquoted, and stored at −20°C for further experiments. PBMCs were isolated from blood collected in K_3_EDTA tubes by density gradient centrifugation. Briefly, PBMCs were isolated by Ficoll-paque plus density gradient (GE) centrifugation at 2000 rpm for 20 min at room temperature. PBMCs were washed 2 times in phosphate-buffered saline and subsequently frozen in liquid nitrogen in 90% fetal bovine serum (FBS, Gibco) with 10% DMSO (Gibco) for further immune profile analysis.

### Pseudovirus-based neutralization assay

A pseudovirus-based neutralization assay was performed as previously described. The lentivirus-based SARS-CoV-2 pseudoviruses express a luciferase reporter gene and bear the S protein from the wild-type strain (Wuhan-Hu-1, Vazyme Biotech Co., Ltd., China). The 50% inhibitory concentration (IC_50_) was defined as the serum dilution at which the relative light units (RLUs) were reduced by 50% compared with the virus control wells. Serial dilutions of heat-inactivated sera (six dilutions in a 4-fold step-wise manner) were incubated with 250 TCID_50_ SARS-CoV-2 pseudoviruses expressing the reporter gene luciferase for 1 h together with the virus control and cell control wells before seeding HEK293T-ACE2 cells in 96-well plates (20,000 cells/well). After 48 h of incubation in 5% CO_2_ at 37°C, the supernatant was removed, and the luminescence was measured using the Luciferase Assay System (Promega Biotech Co., Ltd) according to the manufacturer’s instructions. The IC_50_ values were calculated by generating a three-parameter non-linear regression curve fit in GraphPad Prism v.7.0. The lowest serum dilution tested was 1:4. When no neutralization was observed, we set the pVNT50 value at 2.

### SARS-CoV-2-specific interferon-gamma (IFN-γ) releasing assay (IGRA)

Human IFN-γ ELISpot Kit (U-CyTech, Utrecht, Netherlands) was used for antigen-specific IFN-γ releasing detection. Following the manufacturer’s instruction, a 96-well PVDF plate (Millipore) was conjugated with an anti-IFN-γ antibody overnight at 4°C. 2.5 × 10^5^ PBMCs were incubated with 10 μg/mL SARS-CoV-2 Spike (S) protein (Genscript). Phytohemagglutinin (PHA) (Sigma Aldrich) at a final concentration of 2.5 μg/mL served as a positive control, while the culture medium was a negative control. Stimulated cells were incubated for 20 h at 37°C. Biotin-labelled detection antibody incubated with IFN-γ secreting cells at 4°C overnight. Antibody-labelled plates were incubated with streptavidin-labelled horseradish peroxidase (HRP) at 37°C for 1 h after discarding the supernatant. AEC solution was applied to develop coloration. Plates were then placed in the dark for 30 min. The coloration was terminated by thoroughly rinsing the PVDF membrane with demineralized water. ELISpot Reader (CTL S6 Ultra, Ohio, USA) was used for counting and quantity control after drying out. The number of antigen-specific IFN-γ producing cells was calculated as spot forming units (SFUs) per 2.5 × 10^5^ cells.

### Stimulations for detection of SARS-CoV-2-specific CD4^+^ and CD8^+^ T cells

PBMCs were thawed in RPMI 1640 medium (Gibco) supplemented with 10% FBS (Gibco), and Penicillin Streptomycin (100X, Gibco). Subsequently, 1 × 10^6^ PBMCs were stimulated with SARS-CoV-2 S protein in 200 μl in 96-well U-bottom plates (Corning, PA, USA) in a 5% CO_2_ incubator at 37°C for 20 h. To detect antigen-specific intracellular cytokine levels, GolgiStop (BD Bioscience) was added 14 h after *ex vivo* stimulation. Cells were additionally stimulated with a combination of phorbol 12-myristase 13-acetate (50 μg/ml) and ionomycin (500 μg/ml) as a positive control. After stimulation, cells were stained for phenotypic lymphocyte markers.

### Detection of immune profiles by flow cytometry

The immune profiles of the BBIBP-CorV vaccinated cohort were detected by high-dimensional flow cytometry as described previously [11]. Briefly, cells were stained firstly with surface markers including anti-human CD3-APC-H7 (clone SK7), anti-human CD8-BUV805 (clone OKT4), anti-human CD27-BV786 (clone L128), anti-human CD45RO-BUV563 (clone UCHL1), anti-human CD56-PE-CF594 (clone NCAM16.2), anti-human CD19-BV650 (clone SJ25C1), anti-human CD11c BUV737 (clone B-ly6), anti-human IgD-PE-Cy7 (clone IA6-2), anti-human CD38-APC-R700 (clone HIT2), anti-human IgM-PE (clone G20-127), anti-human CD279 (PD-1)-BUV661 (clone EH12.1), anti-human CD154-BUV615 (clone TRAP1), anti-human CD278-BUV395 (clone DX29), anti-human CD54-BV711 (clone HA58), and fixable viability stain 570 (all from BD Pharmingen) for 30 min in the dark at 4°C. After washing with FACS buffer, cells were fixed with Cytofix/Cytoperm solution for 20 min and permeated with Fix/Perm working solution (BD Bioscience). Cells were then labelled with anti-human IFN-Gma-BV510 (clone B27), anti-human TNF-BV750 (clone MAB11), anti-human IL-2-BV421 (clone 5344.111) (all from BD Pharmingen), anti-human IL-10-APC (clone JES3-19F1, Biolegend) for 30 min in the dark at 4°C. After being washed and resuspended in PBS, cells were acquired with a flow cytometer (BD FACSymphony™). Flow data were analysed using FlowJo software 10.7.1 (FlowJo LLC, BD), and the doublet events were excluded by FSC-A/FSC-H gating.

### Statistical analyses

Prism v.7.0 (GraphPad Software Inc., CA, USA) was used for statistical analyses. Data were represented by a median with an interquartile. Statistical differences were assessed by the Mann–Whitney test for the data with variance homogeneity and by the Kruskal–Wallis test for those with non-variance homogeneity. *P* values < 0.05 were considered statistically significant.

## Results

### BBIBP-CorV vaccination elicits diverse humoral immune responses

**Figure 1.**
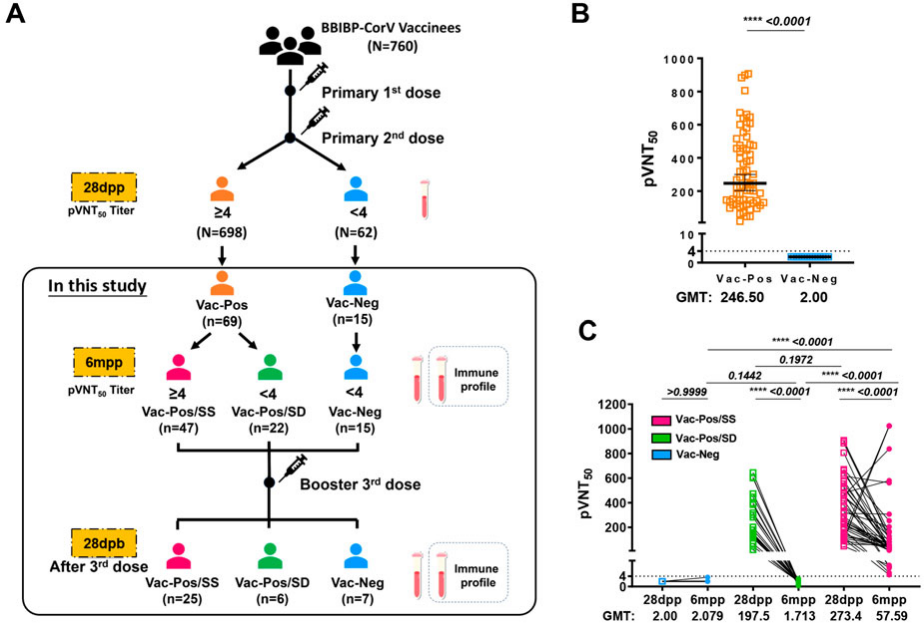
Study design and two-dose BBIBP-CorV vaccination elicit diverse humoral immune responses. (A) Diagram of the study groups. A total of 84 participants were included for the analysis of virus-specific neutralizing antibodies 28 days (28dpp) and 6 months (6mpp) after the completion of the vaccination regimen. Immune profiles were also measured on 6mpp. Forty-seven participants exhibited a sustained level of neutralizing antibodies (Vac-Pos/SS); 22 participants lost their neutralizing antibody level at 6mpp (Vac-Pos/SD); 15 participants never showed the presence of neutralizing antibody (Vac-Neg). (B) Levels of wildtype (WT) spike (S)-specific neutralization antibody were compared between Vac-Pos and Vac-Neg groups at 28dpp. The lowest serum dilution tested was 1:4; undetectable pVNT50 values were set at a pVNT50 of 2. GMTs are indicated above the graph. (C) Levels of WT S-specific neutralizing antibody were analysed early (28dpp) and late (6mpp) after the 2-dose vaccination. Lines and bars indicated median with interquartile range (IQR). *P* value determined by unpaired *t*-test (B) or by paired *t*-test (C).

A total of 760 health care workers (HCWs) from Shanghai Ruijin Hospital have been previously enrolled in a study of vaccine efficacy evaluation after two-dose BBIBP-CorV vaccination [10]. We collected the sera on day 28 (28dpp) after the second dose to evaluate S-nAb levels by pVNT50 titres according to a pseudovirus-based neutralization assay. Accordingly, 698 HCWs (91.8%) who had S-nAb pVNT50 titres more than 4 were designed as the Vac-Pos group, whereas 62 HCWs (8.2%) had pVNT50 titres less than 4 as the Vac-Neg group (Figure 1(A)). We further followed up 84 HCWs voluntarily among which 69 belonged to the Vac-Pos group and 15 belonged to the Vac-Neg group for 6–9 months (6mpp) after the second-dose BBIBP-CorV vaccination. Based on S-nAb titres at 6mpp, the Vac-Pos group could be further divided into the Vac-Pos/SS group (*n* = 47, 70.1%) whose pVNT50 titres were more than 4, and the Vac-Pos/SD group (*n* = 22, 31.9%) whose pVNT50 titres were less than 4, while pVNT50 titres of the Vac-Neg group (*n* = 15) remained less than 4 (Figure 1 (A)). Characteristics of all the participants enrolled in this study are summarized in Table S1. In addition, all the HCWs received booster (third-dose BBIBP-CorV) vaccination.

It was obvious that at 28dpp after the second dose vaccination the average pVNT50 values of S-nAb in the Vac-Pos (*n* = 69) group was 246.5 geometric mean neutralization titre (GMT) (95% Confidence intervals (CI): 202.8–299.6), whereas Vac-Neg (*n* = 15) exhibited S-nAb deficiency (Figure 1(B)). There was no significant difference in GMT values between the Vac-Pos/SS group (273.4, 95% CI: 219–341.4) and the Vac-Pos/SD group (197.5, 95% CI: 132.7–294.1) (*p *= 0.1574) at 28 dpp. At 6mpp both Vac-Pos/SS and Vac-Pos/SD groups exhibited the attenuation of S-nAb pVNT50 titres with different trends. GMT values of the Vac-Pos/SS group decreased from 273.4 to 57.59 (95% CI: 37.72–87.95), whereas those of the Vac-Pos/SD group decreased from 197.5 to 1.713 (95% CI: 1.392–2.107) (Figure 1(C)). Therefore, apart from a small proportion of the vaccinees with *de novo* S-nAb deficiency (the Vac-Neg group), Vac-Pos group vaccinees experienced dramatic attenuation of S-nAb after 6 months of two-dose vaccination among which 31.9% (22/69) became seronegative, which indicates the impairment of long-term maintenance of S-nAb after two-dose vaccination.

### Comparable T cell responses induced by 2-dose BBIBP-CorV

**Figure 2.**
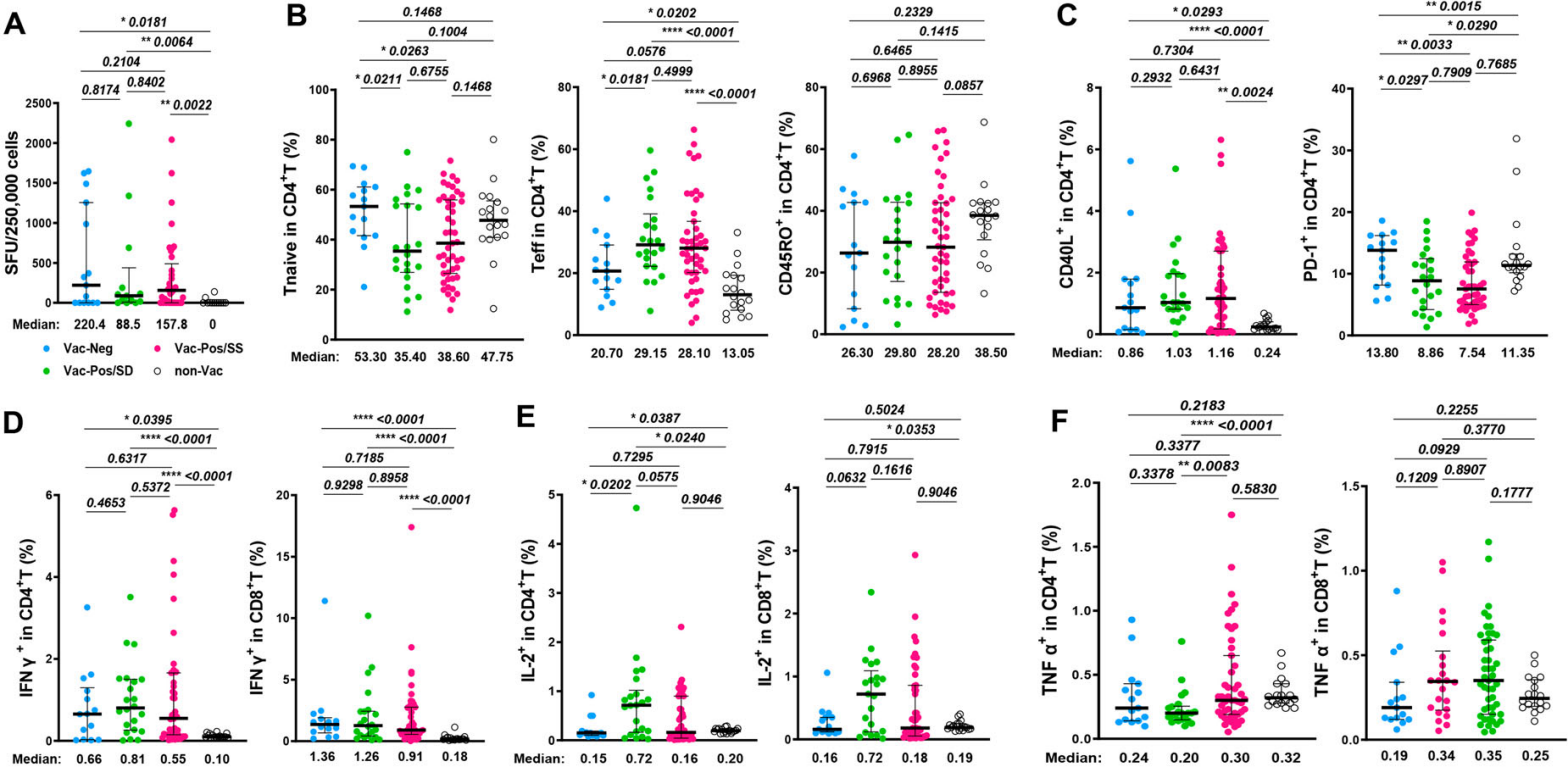
Viral-specific T cell response in vaccinees after two-dose BBIBP-CorV vaccination at 6 months. (A) Interferon-gamma (IFNg) releasing cell numbers in serosustain (Vac-Pos/SS, *n* = 37) and seronegative (Vac-Neg, *n* = 15). (B) Comparisons of the percentages of naïve T (Tnaive), effector T cells (Teff), and CD45RO^+^ memory CD4^+^ T cells among Vac-Neg, Vac-Pos/SS, Vac-Pos-SD, and Non-Vac groups. (C) Percentages of CD40L and PD-1 expressing CD4^+^ T among Vac-Neg, Vac-Pos/SS, Vac-Pos-SD, and Non-Vac groups. (D–F) Comparisons of WT strain S protein-specific IFNγ (D), IL-2 (E), and TNFα (F) producing CD4^+^ T cells among Vac-Neg, Vac-Pos/SS, Vac-Pos-SD, and Non-Vac groups. Individuals without vaccine injection (non-Vac) were taken as the control group. Data were represented as median with interquartile range (IQR). *P* value was calculated by unpaired *t*-test.

Since HCWs from both Vac-Neg and Vac-Pos/SD groups exhibited the deficiency in S-nAb responsiveness to BBIBP-CorV immunization at 6mpp, whether this was due to the defects in antigen-specific T cell responses was further investigated. When we stimulated PBMCs from either Vac-Pos or Vac-Neg individuals with SARS-CoV-2 S protein at 6mpp, we found that the spot forming units (SFUs) of S-specific interferon-gamma (IFN-γ) releasing cells were comparable between Vac-Pos/SS (157.8 SFUs, interquartile (IQR): 0–489.1) and Vac-Neg (220.4 SFUs, IQR: 0–1254) subjects (*p* = 0.2104), as well as between Vac-Pos/SS and Vac-Pos/SD groups (88.5 SFUs, IQR: 8.898–439) (*p *= 0.8402) whereas SFUs from the non-Vac group were undetectable with a significant difference when compared to the other three groups (Figure 2(A)).

Considering the important roles of CD4^+^ T cells in the induction of humoral immune responses, we further compared the profiles of CD4^+^ T cells among Vac-Neg, Vac-Pos/SD, or Vac-Pos/SS groups by flow cytometry (see gating strategies in Figure S1). Whilst no difference in the frequencies of total CD4^+^ T cells among three groups (Figure S1B), Vac-Neg vaccinees showed higher proportions of CD45RO^-^CD27^+^ naïve CD4^+^ T cells (T_N_) when compared to Vac-Pos/SS (*p *= 0.0263) or Vac-Pos/SD (*p *= 0.0211) subjects and lower proportions of CD45RO^-^CD27^-^ effector CD4^+^ T cells (T_eff_) (*p *= 0.0576 to Vac-Pos/SS, *p *= 0.0181 to Vac-Pos/SD), which were similar to those in the non-Vac group. There was no difference in the frequencies of CD4^+^ T_N_ cells (*p *= 0.6755), CD4^+^ Teff cells (*p* = 0.4999), and CD45RO^+^ memory CD4^+^ T cells (T_M_) (*p* = 0.8955) between Vac-Pos/SS and Vac-Pos/SD groups (Figure 2(B)). We also detected certain costimulatory molecules on CD4^+^ T cells involved in B cell activation and differentiation including CD40L and programmed death-1 (PD-1). CD40L is inducibly expressed on activated CD4^+^ T cells to provide costimulatory signals for B cell activation, whereas PD-1 on activated T cells exerts as an inhibitory regulator for CD4^+^ T cell function [12,13]. Although we have detected diverse S-nAb levels in Vac-Neg, Vac-Pos/SS, and Vac-Pos/SD groups at 6mpp, the percentages of CD40L-expressing CD4^+^ T cells among three groups were comparable, while all were higher than those in the non-Vac group (Figure 2(C), left panel). On the other hand, both Vac-Pos/SS and Vac-SS/SD individuals exhibited significantly lower percentages of PD-1-expressing CD4^+^ T cells in the periphery than the Vac-Neg group (*p* = 0.0033 to Vac-Pos/SS, *p* = 0.0297 to Vac-Pos/SD) (Figure 2(C), right panel). Therefore, CD4^+^ T cells in the periphery of both Vac-Neg and Vac-Pos groups displayed similar activation levels no matter whether antibody responses maintain or attenuate at 6mpp. Downregulation of PD-1 expression on CD4^+^ T cells in both Vac-Pos/SS and Vac-Pos/SD groups as compared to Vac-Neg group more likely implies the associations between the reinforcement of CD4^+^ T cells and the induction of S-nAb after two-dose BBIBP-CorV.

We further determined S protein-specific cytokine production in CD4^+^ and CD8^+^ T cells for functional assessment. Upon S protein *ex vivo* stimulation, Vac-Neg, Vac-Pos/SS, and Vac-Pos/SD groups exhibited similar percentages of IFN-γ producing CD4^+^ and CD8^+^ T cells in the periphery at 6mpp, whereas all were higher than the non-Vac group (Figure 2(D)), which was consistent with the ELISPOT results. The percentages of IL-2-producing CD4^+^ and CD8^+^ T cells in the Vac-Pos/SD group were slightly elevated when compared to either Vac-Neg or Vac-Pos/SS group (Figure 2(E)). No obvious differences were detected in S protein-specific TNF-α^+^ CD4^+^ and CD8^+^ T cells among all four groups (Figure 2(F)). Collectively, S-specific IFN-γ responsiveness and T cell responses at 6mpp after the second dose of BBIBP-CorV are comparable no matter that S-nAb is absent *de novo* (Vac-Neg group) or waned (Vac-Pos/SD group) after 6mpp, indicating fewer variations of T cell immunoreactivity after BBIBP-CorV vaccination.

### Circulating B cell profiles before and after booster vaccination

**Figure 3.**
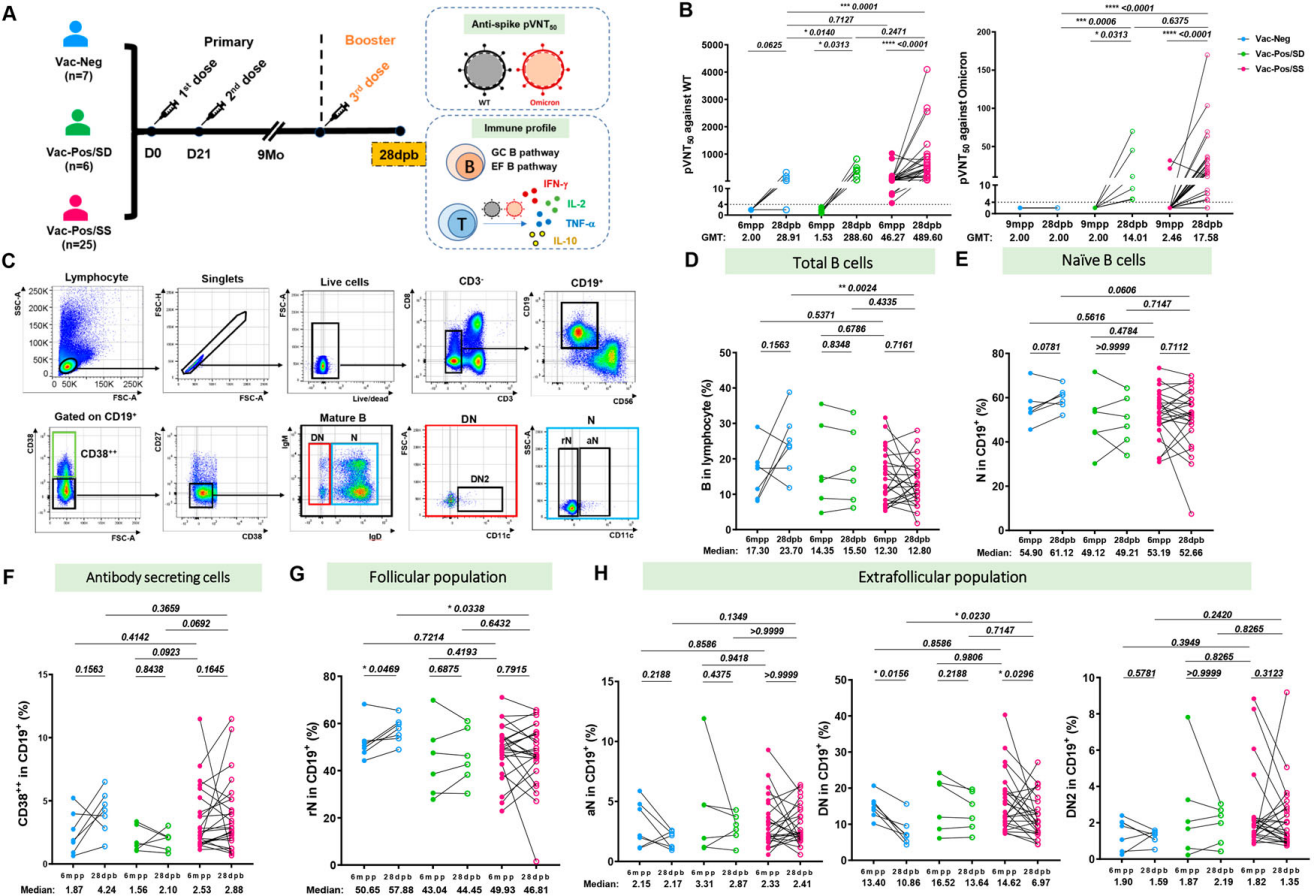
B cell differentiation profiles before and after booster BBIBP-CorV vaccination. (A) Diagram of the booster regimen. *n* = 25 Vac-Pos/SS, *n* = 6 Vac-Pos/SD, *n* = 7 Vac-Neg were included in the immune profile analysis at 28 days after the booster programme (28dpb). All participants were boosted at 9 months after the completion of the 2-dose BBIBP-CorV vaccination. (B) Comparison of neutralizing antibody levels against WT (left) and Omicron (right) strains in participants who received booster vaccination before (6 mpp) and after booster vaccination (28 dpb). GMTs are indicated above the graph. The dotted line indicates the lowest serum dilution (1:4). (C) Gating strategy of flow cytometry data for different B cell subpopulations. (D-E) Percentages of total CD19^+^ B cells and naïve B cells in Vac-Neg, Vac-Pos/SS, and Vac-Pos-SD groups before (6mpp) and after (28dpb) booster vaccination. (F-H) Frequencies of CD38^+^ (F), resting naïve (rN) B cells (G) and extrafollicular population (H), including activated naïve (aN), CD27^-^IgD^-^ (double negative, DN), and CD11c^+^ DN (DN2) cells in CD19^+^ B cells before (6mpp) and after (28dpb) booster vaccination. Data were represented as median with interquartile range (IQR). *P* value was determined by paired *t*-test.

Booster immunization (third injection) is highly recommended to restore the efficacy of BBIBP-CorV vaccines [14]. Owing to the diverse S-nAb profiles among Vac-Neg, Vac-Pos/SS, and Vac-Pos/SD groups after two-dose vaccination, we have tracked S-nAb as well as T cell responses in partial Vac-Neg (*n* = 7), Vac-Pos/SS (*n* = 25), and Vac-Pos/SD (*n* = 6) vaccinees who finished booster immunization at 9 months after regular two-dose BBIBP-CorV primary vaccination. Sera and PBMCs were collected at 28 days post booster (28dpb) (Figure 3(A), Table S2). It was obvious that booster vaccination elicited a rapid increase in S-nAb levels in all three groups. For instance, 5 out of 7 participants in the Vac-Neg group displayed the induction of S-nAb. The GMT pVNT_50_ values of Vac-Neg group reached 28.91 (95%CI: 4.352–192) (*p *= 0.0210 to those before booster vaccination), which was comparable to pVNT_50_ values of the Vac-Pos group at 6mpp (GMT: 23.92, 95% CI 12.19–46.96) (*p *= 0.9352) (Figure S2). Moreover, both Vac-Pos/SS and Vac-Pos/SD vaccinees showed a dramatic increase in S-nAb titres after booster vaccination as well where GMT values of Vac-Pos/SS vaccinees reached 489.6 from 46.27 at 6 mpp (*p *= 0.0313) and those of Vac-Pos/SD vaccinees increased from 1.532 to 288.6 at 6mpp (*p *< 0.0001). When we calculated the seroconversion rates at 28dpb which was defined as a change of titres from seronegative at 6mpp to seropositive at 28dpb with a four-fold increase in pVNT50 titres, 71.43% (5/7) of the participants showed the seroconversion within the Vac-Neg group, 100% (6/6) in the Vac-Pos/SD group and 76% (19/25) in the Vac-Pos/SS group (Figure 3(B), left panel). Due to the prevalence of Omicron recently, we have also determined the neutralizing activity of the sera before and after booster vaccination in partial follow-up vaccinees. It was obvious that with seronegative activity to Omicron variant before booster immunization in all individuals we detected, both Vac-Pos/SD (5/5) and Vac-Pos/SS (23/25) groups exhibited an increase in S-nAb pVNT_50_ values against Omicron variant nevertheless which were dramatically lower than those to WT strain in both groups (Figure 3(B), right panel).

It is well acknowledged that B cells differentiate into terminal antibody-secreting cells mostly through germinal centre (GC) pathways and under certain circumstances by extrafollicular B cell (EFB) differentiation pathways [15], which is also correlated with nAb responses upon SARS-CoV-2 infection [16]. Due to the dramatic increase in S-nAb titres after booster vaccination, we, in parallel, analysed peripheral B cell differentiation subsets related to GC and EFB pathways before and after booster vaccination, including total B cells (CD19^+^), naïve (N, CD38^-^CD27^-^IgD^+^ CD19^+^) B cells, CD38^++^CD19^+^ B cells as terminally differentiated B cell subset, and double negative (DN, CD38^-^CD27^-^IgD^-^CD19^+^), resting naïve (rN, CD38^-^CD27^-^IgD^+^ CD11c^-^CD19^+^), activated naïve (aN, CD38^-^CD27^-^IgD^+^ CD11c^+^ CD19^+^), and DN2 (CD38^-^CD27^-^IgD^-^CD11c^+^ CD19^+^) cells for EFB pathway (Figure 3(C)). We found that booster vaccination did not affect the frequencies of total CD19^+^ B cells (Figure 3(D)) and naïve B cells (Figure 3(E)) among Vac-Neg, Vac-Pos/SS, and Vac-Pos/SD groups before and after booster vaccination. Interestingly, in the Vac-Neg group we detected an increase in the percentages of antibody-secreting CD38^+^ CD19^+^ B cells after booster vaccination (Figure 3(F), blue dots), which was consistent with the appearance of anti-S antibodies in the periphery. Moreover, the percentage of GC-related rN B cell subset was increased (*p* = 0.0496) (Figure 3(G), blue dots) and those of EFB-related DN subsets exhibited a decline (*p* = 0.0156) in the Vac-Neg group (Figure 3(H), middle panel, blue dots) after booster vaccination. There were no significant differences in the percentages of total B cells and B cell differentiation subsets between Vac-Pos/SS and Vac-Pos/SD groups (Figure 3(D–H), green and red dots) before and after booster vaccination. Therefore, with the enhancement of S-nAb activity after booster vaccination, we have observed an increase in terminal differentiation in Vac-Neg vaccinees with more GC B cell subset and declined EFB cells after booster vaccination. However, this tendency is not detectable in Vac-Pos vaccinees after booster vaccination whose S-nAbs have been induced already during primary vaccination.

### Booster vaccination induces comparable T cell responses against WT and Omicron variant

**Figure 4.**
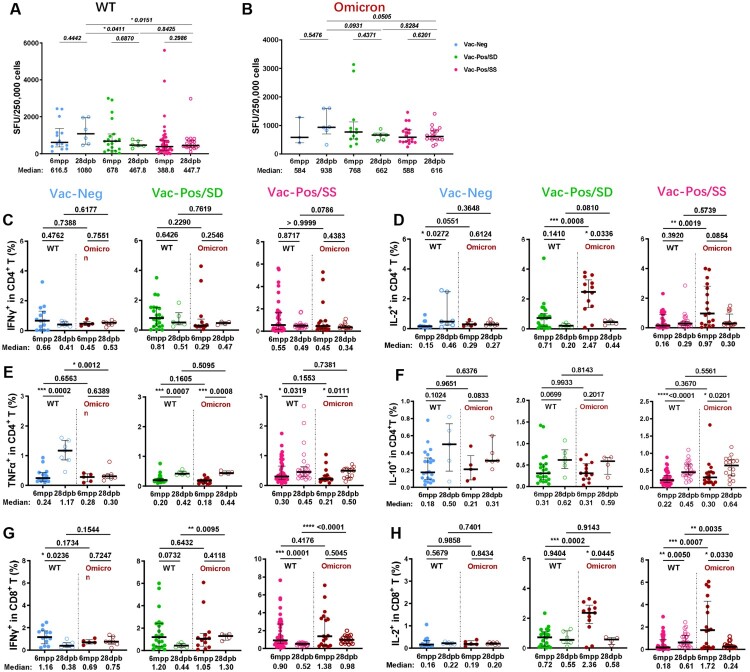
S protein-specific cellular immune responses after BBIBP-CorV booster vaccination. (A–B) WT (A) and Omicron (B) S protein-specific IFN-γ releasing cell numbers in Vac-Neg, Vac-Pos/SS, and Vac-Pos/SD groups before (6mpp) and after (28dpb) booster vaccination. (C–F) Comparisons of WT and Omicron S protein-specific IFN-γ (C), IL-2 (D), TNF-α (E), and IL-10 (F) producing CD4^+^ T cells before (6mpp) and after (28dpb) booster vaccination in Vac-Neg, Vac-Pos/SS, and Vac-Pos/SD groups. (G–H) Comparisons of WT and Omicron S protein-specific IFN-γ (G) and IL-2 (H) producing CD8^+^ T cells before (6mpp) and after (28dpb) booster vaccination in Vac-Neg, Vac-Pos/SS, and Vac-Pos/SD groups. Data were represented as median with interquartile range (IQR). *P* value was determined by paired *t*-test.

WT and Omicron S protein-specific T cell responses were detected at 28dpb as well. Along with the increase in S-nAb levels, Vac-Neg vaccinees exhibited more frequencies of antigen-specific IFN-γ-producing cells at 28dpb (Median SFUs: 1080 for WT) when compared with those at 6mpp (Median SFUs: 616.5 for WT) (Figure 4(A), blue dots), both of which were extraordinarily higher than those in Vac-Pos/SS (Median SFUs: 447.7) (*p *= 0.0151) and Vac-Pos/SD (Median SFUs: 467.8) (*p *= 0.0411) (Figure 4(A), green and red dots). The tendency of antigen-specific IFN-γ producing cells was similar to Omicron S protein immunoreactivity. Vac-Neg vaccinees exhibited increased frequencies of IFN-γ-producing cells after booster vaccination (Median SFUs: 938) as compared to those at 6mpp (Median SFUs: 584), which was also the highest among Vac-Neg, Vac-Pos/SD, and Vac-Pos/SS groups (Figure 4(B), blue dots). IFN-γ-producing cells upon S protein stimulation from either WT or Omicron isolates in Vac-Pos/SS and Vac-Pos/SD groups remained at similar levels at 28dpb when compared to those at 6mpp (Figure 4(A and B), green and red dots).

We further analysed the capacity of cytokine production in CD4^+^ and CD8^+^ T cells upon S protein stimulation from either WT or Omicron after booster vaccination among three groups. The percentages of IFN-γ producing CD4^+^ T cells were comparable before and after booster vaccination among Vac-Neg, Vac-Pos/SD, and Vac-Pos/SS groups (Figure 4(C)) as well as the percentages of IL-2-producing CD4^+^ T cells (Figure 4(D)). There was an increase in TNF-α production in CD4^+^ T cells from three group vaccinees after booster vaccination (Figure 4(E)). No difference was observed in IL-10 producing CD4^+^ before and after booster vaccination against WT and Omicron S proteins among the three groups as well (Figure 4(F)). For CD8^+^ T cells, there was a decrease in IFN-γ-producing CD8^+^ T cells upon S protein stimulation after booster vaccination among the three groups (Figure 4(G)), whereas no difference was in IL-2-producing CD8^+^ T cells (Figure 4(H)). From March to June 2022, the Omicron epidemic was prevailing regionally in China including in Shanghai. Among all 760 participants who were previously enrolled in our vaccine efficacy evaluation study, 16 participants (2 participants of the Vac-Pos/SD group in this study) were infected by Omicron (BA.2) variants without any severe cases, regardless of their diverse neutralizing antibody levels before infection. Collectively, these results further demonstrate that BBIBP-CorV-induced specific T cell responses are more stable than B cell responses and may play a role in the prevention of severe COVID-19 infection even encountering the Omicron variant.

## Discussion

Induction of nAb after SARS-CoV-2 vaccines is one of the key criteria to evaluate vaccine efficacy. Although all emergently authorized vaccines are demonstrated to induce the rapid generation of S-nAb after one- or two-dose vaccination within one month, cohort follow-up studies also report the decline of S-nAb after 6–9 months, including two-dose BBIBP-CorV vaccination [10,17], which warrants great concerns of long-term protections. In the present study, we have demonstrated that antigen-specific T cell responses were comparable no matter whether S-nAb levels declined or not after 6 months in vaccinees receiving two-dose BBIBP-CorV vaccination. Booster vaccination mostly restored S-nAb levels, whereas antigen-specific T cell responses remained at stable states as well. More interestingly, T cell responses targeting the Omicron variant were similar to those targeting WT isolate although antibody neutralizing activity to Omicron was dramatically lower when compared to that to WT isolate after booster vaccination [9,18]. Combined our and other studies on specific B cell and T cell responses induced by different types of SARS-CoV-2 vaccines , it is apparently demonstrated that viral-specific T cell responses are inclined to remain stable although B cell responses are more variable with the elongation of vaccination.

Long-term maintenance of anti-viral nAb responses elicited by SARS-CoV-2 vaccines is recognized as the main factor to prevent the progression of disease severity but is not sufficient to resist re-infection [19–21]. We have reported the decline of neutralization potency against SARS-CoV-2 after the second dose of BBIBP-CorV at 6mpp [10]. Apart from a small proportion (about 10%) of individuals featuring *de novo* absence of S-nAb after two-dose vaccination in the Vac-Neg cohort, we have observed the dramatic decline of S-nAb levels after 6 months in Vac-Pos vaccinees 33% of which have become S-nAb negative. However, 28 days after booster vaccination we have observed rapid restoration of S-nAb in 100% of Vac-Pos/SD vaccinees whose S-nAb levels have decayed dramatically at 6mpp. Moreover, S-nAb was also induced in most of Vac-Neg individuals, indicating the importance of booster vaccination to establish an extensive humoral immunity barrier against SARS-CoV-2.

Vaccine-induced T cell responses targeting S protein occur as early as 1 month after immunization and maintain at least for 6 months reported in multiple SARS-CoV-2 vaccine investigations [22]. Herein in contrast to diverse S-nAb levels after 6 months of two-dose BBIBP-CorV vaccination among Vac-Neg, Vac-Pos/SS, and Vac-Pos/SD groups, we have observed comparable S protein-specific T cell responses with similar levels of IFN-γ release and cytokine production as well as CD40L^+^ CD4^+^ cells. This is consistent with the study from the mRNA vaccine that although the S-specific binding antibody level decreased after 6 months of the vaccination, virus-specific T cells were still detectable [8]. In addition, we observed that individuals in the Vac-Neg group with *de novo* deficiency in S-nAb exhibited higher percentages of naïve CD4^+^ T cells and less frequency of effector CD4^+^ T cells (Figure 2(B)) whereas CD4^+^ T cells expressed similar levels of CD40L when compared to the Vac-Pos group. PD-1 expression on CD4^+^ T cells was higher in the Vac-Neg group than that in the Vac-Pos, which is consistent with previous work that PD-1 blockade could enhance humoral immunity [23]. These results suggest that in the Vac-Neg group although CD4^+^ T cells are activated upon BBIBP-CorV vaccination they might provide fewer costimulatory signals for B cell terminal differentiation. The mechanisms of *de novo* deficiency in S-nAb need to be further investigated.

A booster vaccination is the most efficient way to restore S-nAb responses in multiple vaccination strategies as well as BBIBP-CorV [24,25]. In our study, S-nAb levels in Vac-Pos/SS and Vac-Pos/SD groups are even higher at 28 days after booster vaccination than those at 28 days after the second dose of BBIBP-CorV vaccination, indicating the presence and high efficiency of BBIBP-CorV-induced memory B cell immunity. Even in Vac-Neg groups, we have also detected the generation of S-nAb. This also indicates that although around 10% of the individuals could not generate sufficient anti-viral humoral immunity after two-dose BBIBP-CorV primary vaccination, booster vaccination would help to establish anti-viral humoral immunity with similar efficiency. Compared to Vac-Pos/SS and Vac-Pos/SD groups, the levels of S-nAb is much lower. It will be interesting whether using S protein-derived vaccines or additional booster vaccination could restore the S-nAb barrier with more efficiency in Vac-Neg subjects, which is worthy of further investigations.

Based on the analysis of B cell differentiation subsets before and after booster vaccination, we have observed that individuals in the Vac-Neg group exhibited a decrease in activated naïve B cells and DN B cells before and after booster vaccination along with an increase in GC-related CD38^++^ B cells. Activated naïve B cells and DN B cells are two important components in the EFB pathway [26], while antibody-secreting cells from GC pathways express a high level of CD38 [27]. It has previously been reported that limited longevity of neutralizing antibodies in severe COVID-19 patients is associated with EFB cell response [16]. Since classical B cell termination and differentiation to plasma cells through the GC pathway are largely dedicated to inducing high-affinity and long-life memory B cell responses, our result implies that booster vaccination might trigger a shift from the EFB pathway to GC response for the generation of neutralizing antibody in the Vac-Neg group, which might reflect B cell differentiation at the early stages of BBIBP-CorV vaccination. Whether B cell differentiation through EFB pathways is related to limited maintenance of S-nAb will be very helpful to design long-term SARS-CoV-2 vaccines.

We have also detected humoral and cellular immunoreactivity targeting recently prevalent Omicron-derived S protein after booster vaccination. Although antibody neutralizing activity to Omicron was reduced dramatically, Omicron S protein-specific T cell responses were comparable to WT S protein. According to genome sequences of receptor binding domain (RBD) from WT and Omicron, there are nearly 31 nucleotide variations in RBD. This is associated with immune escape from S-nAb induced by WT isolate-derived vaccines [28] and also might affect antigen-specific cellular responses. However, according to our results and others [29–31], WT isolate-derived vaccines induced T cell responses with similar immunoreactivity to Omicron S protein. This might be due to the redundancy of T cell recognition to viral peptide, which is partially supported by TCR repertoire sharing between WT and SARS-CoV-2 VOC infection [19,22,32]. More remarkably, we have observed apparently protection of BBIBP-CorV vaccination for the reduction of COVID-19 severity even upon Omicron infection, consisting of previous reports about viral-specific T cell responses induced by other types of SARS-CoV-2 vaccines [33,34].

Although we have profiled antibody neutralizing activity and S protein-specific T cell responses at 6 months after two doses of BBIBP-CorV vaccination and 28 days after booster vaccination, our relatively small sample size and the large loss of follow-up between 28 days and 6 months after vaccination may limit further information on vaccine-induced immunity. Moreover, whether similar levels of T cell responses to WT and Omicron strains are due to cross-reactivity or epitope spreading needs to be further defined.

Collectively, with a well-designed prospective sample collection, we demonstrate that BIBP-CorV vaccination induces equivalent anti-SARS-CoV-2 specific T cell responses no matter whether neutralizing antibody responses are present or absent. After booster vaccination, antibody neutralizing activity to WT isolate is restored with less efficacy to Omicron, whereas T cell responses to WT and Omicron isolates are still comparable. This was partially consistent with the good prevention of severe infection during the Omicron epidemic. Therefore, unlike fluctuated viral-specific humoral immune responses T cell responses induced by the BIBP-CorV vaccine are more stable at different stages of vaccination.

## Supplementary Material

Supplemental Material
