# Supplementary material for: Comparable antigen-specific T cell responses in vaccinees with diverse humoral immune responses after the primary and booster BBIBP-CorV vaccination
Source: Emerg Microbes Infect. 2022 Oct 26;11(1):2474–84. doi: 10.1080/22221751.2022.2130101 (PMC9621266; doi:10.1080/22221751.2022.2130101)
Supplement: Supplemental Material [file TEMI_A_2130101_SM7827.docx]

|  | **N** | **Female (N, %)** | **Age (Median years, interquartile range [IQR])** |
| --- | --- | --- | --- |
| **Vac-Neg** | 15 | 14 (93.33%) | 38 (28-44) |
| **Vac-Pos** | 69 | 54 (78.26%) | 43 (36-50) |
| **Vac-Pos/SD** | 22 | 19 (86.36%) | 42 (36-48) |
| **Vac-Pos/SS** | 47 | 35 (74.47%) | 44 (32-52) |

Supplementary Materials

**Table S1. Participant information in the primary vaccination cohort.**

**Table S2. Participant information in the boost vaccination cohort.**

|  | **N** | **Female (N, %)** | **Age (Median years, interquartile range [IQR])** |
| --- | --- | --- | --- |
| **Vac-Neg** | 7 | 6 (85.71%) | 41 (28-46) |
| **Vac-Pos** | 31 | 20 (64.52%) | 44 (39-52) |
| **Vac-Pos/SD** | 6 | 3 (50.00%) | 43 (38.75-46.25) |
| **Vac-Pos/SS** | 25 | 17 (68.00%) | 44 (40-54) |


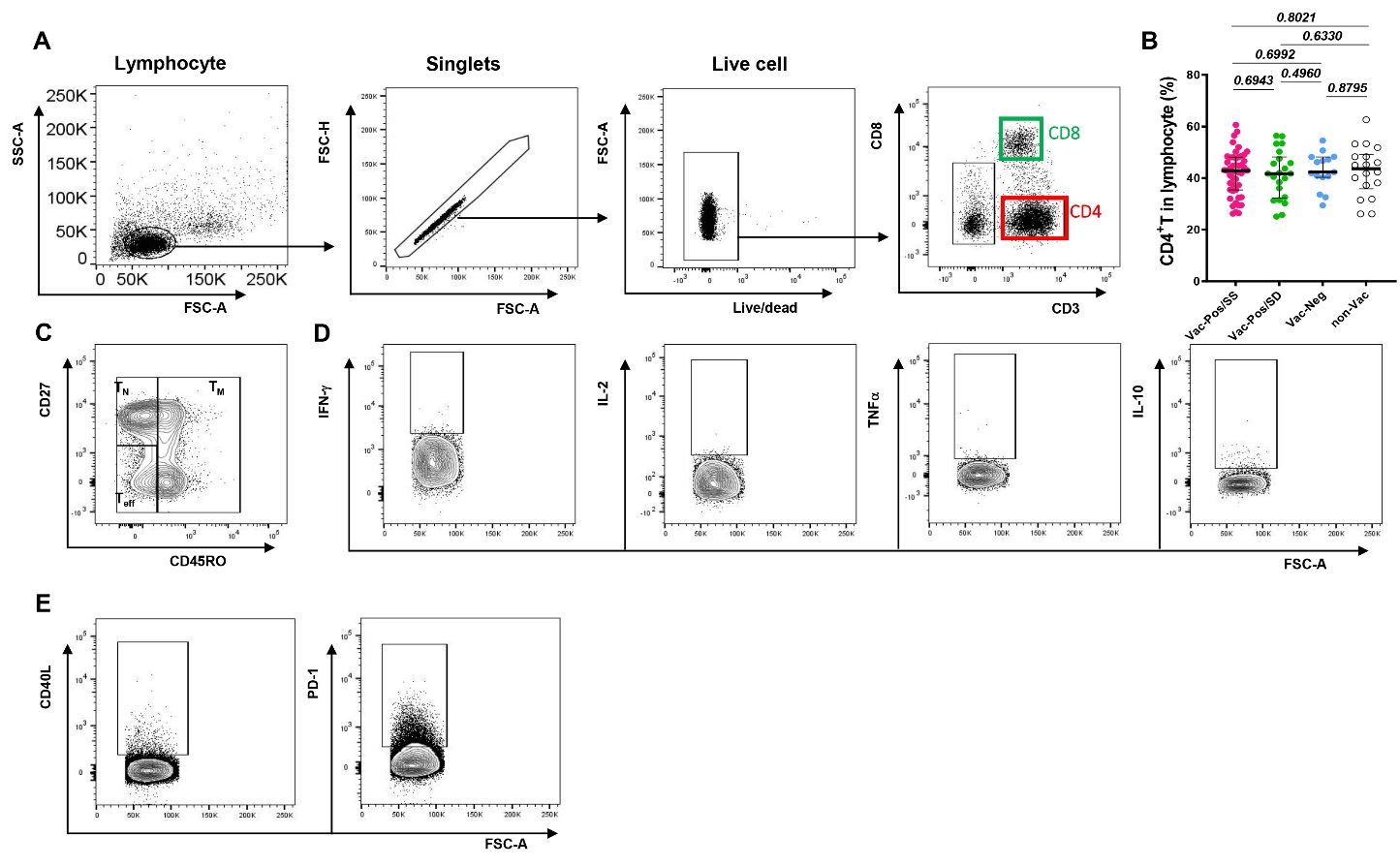


**Figure S1. Gating Strategies of T cell immune profiling.** (A) Lymphocytes were gated from peripheral blood mononuclear cells (PBMCs), and singlets were defined by FSC-H and FSC-A gating. Live cells can not be labeled by fixable viability stain 570 (BD Bioscience). CD4+T and CD8+T cells were classified by CD3 and CD8 gating. (B) Frequency of CD4+T cells in lymphocytes were analyzed and there were no difference between Vac-Pos/SS, Vac-Pos/SD, Vac-Neg, and non-Vac groups. (C) CD27 vs CD45RO gating strategies identified CD45RO-CD27+ naïve T cells (TN), CD45RO+ memory T cells (TM), and CD45RO-CD27- effector T cells (Teff). (D) Cytokines expression upon SARS-CoV-2 S1 protein in vitro stimulation in CD4+T or CD8+T cells including IFN-g, IL-2, TNFa, or IL-10. (E) Co-stimulatory molecules expression on CD4+T cells including CD40L and PD-1.


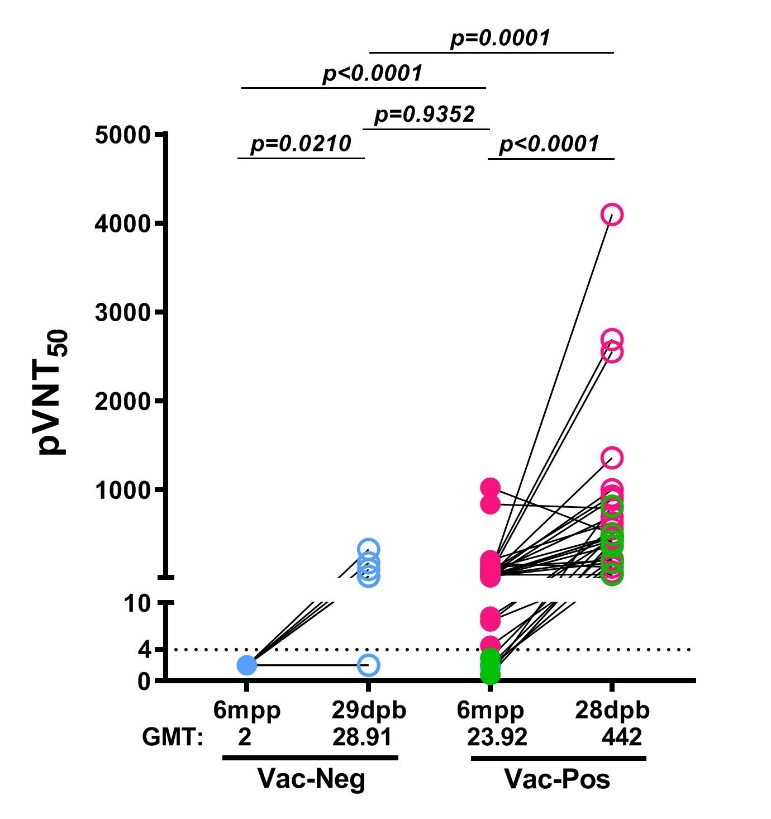


**Figure S2. Comparison of neutralizing antibody levels against wildtype strain of SARS-CoV-2 pseudovirus in Vac-Neg and Vac-Pos groups before and after booster BBIBP-CorV vaccination.**
